# ROTAVI: simultaneous left main rotablation and transcutaneous aortic valve implantation in calcified coronaries and severe aortic stenosis – a case report

**DOI:** 10.1093/ehjcr/ytaa196

**Published:** 2020-08-23

**Authors:** Himanshu Gupta, Navjyot Kaur, Yashpaul Sharma, Parag Barwad

**Affiliations:** Department of Cardiology, PGIMER, Chandigarh 160012, India; Department of Cardiology, PGIMER, Chandigarh 160012, India; Department of Cardiology, PGIMER, Chandigarh 160012, India; Department of Cardiology, PGIMER, Chandigarh 160012, India

**Keywords:** Complex LM PCI, Rotablation, Single setting, TAVR, Case report

## Abstract

**Background:**

There is a high incidence of calcified coronary artery disease in patients with severe valvular aortic stenosis (AS). With transcutaneous aortic valve replacement (TAVR) as one of the promising options for severe AS in high and intermediate surgical risk patients; we will encounter more and more patients who will require both complex percutaneous coronary intervention (PCI) with rotablation (RA) and TAVR. The timing of PCI in patients undergoing TAVR; however remains indecisive. Due to the complexity of procedures and the risks involved, very few cases of concomitant TAVR and coronary RA have been reported so far.

**Case summary:**

Seventy-five years old high surgical risk female had severe AS with calcified left main (LM) distal and ostial left anterior descending (LAD) artery lesion. Successful PCI with RA to LM-LAD lesion was done followed by uneventful transfemoral TAVR in the same setting.

**Discussion:**

This is probably one of the very few cases reported where PCI to LM with RA and TAVR was done successfully in the same setting. Since the calcified lesion was focal and left ventricular ejection fraction of the patient was normal, we went ahead with PCI without prior balloon dilatation of aortic valve (BAV) which was a deviation from the prior reported cases, where BAV was performed prior to complex PCI to improve the cardiac output. We herein discuss our case and thoughts about concomitant complex PCI and TAVR.

**Learning points**

- With more experience in transcutaneous aortic valve replacement (TAVR) and positive results of SURTAVI and PARTNER II, a large number of patients will now be candidates for concomitant percutaneous coronary intervention (PCI) and TAVR.
- In carefully selected patients, complex PCI requiring rotablation can be done in the same setting as TAVR.
- Till more data are available, the timing of PCI in patients planned for TAVR needs to be individualized.

## Introduction

Patients with severe valvular aortic stenosis (AS) often have concomitant calcified coronary artery disease (CAD).^1^ Advanced age, frailty, and co-morbidities make these individuals a very high risk for surgery. The less invasive transcutaneous aortic valve replacement (TAVR) with percutaneous coronary intervention (PCI) remains a viable option. However, rotablation (RA) is usually required for lesion preparation in such calcified arteries. Very few cases have been reported where PCI with RA was combined with TAVR in the same setting.^2^^,^^3^ We herein present our case where we successfully performed PCI to heavily calcified left main (LM) with the help of RA, followed by TAVR in the same setting.

## Timeline

**Table:** 

Six months prior to presentation	75-year-old frail female with insulin-dependent diabetes mellitus (DM), hypertension (HTN), and chronic kidney disease (CKD) Stage III; symptomatic with New York Heart Association Class III dyspnoea
One month prior to admission	Evaluation: Severe valvular aortic stenosis (AS)
	Calcified left anterior descending (LAD) disease
	Refused surgery due to high STS score and EUROSCORE
	Evaluation for the feasibility of transfemoral (TF) Transcutaneous aortic valve replacement (TAVR)
	Computed tomography aortogram: Suitable for TF-TAVR
During current admission	Procedure: Underwent uneventful percutaneous coronary intervention (PCI) to left main LAD with rotablation (RA) with minimal contrast followed by TF-TAVR for severe AS in the same setting No balloon dilatation of aortic valve done prior to PCI No left ventricular assist device used Normal left ventricular systolic ejection fraction, focal calcified lesion, and expertise in RA guided us to do PCI first without dilating the valve
Discharge	Discharged on Day 3 of the procedure with:
	No aortic regurgitation
	No local site complications
	No worsening of renal functions

## Case presentation 

**Figure 1:**
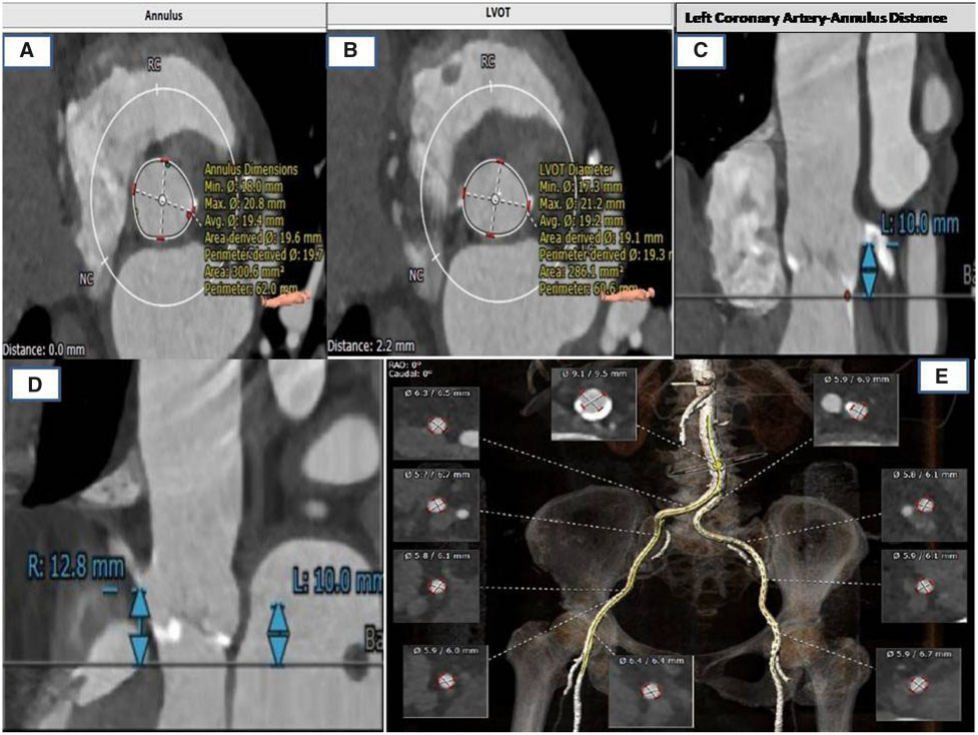
Computed tomography images of aortic annulus and left ventricular outflow tract (*A, B*). Left coronary ostial-annular distance (*C*). Right coronary ostial-annular distance (*D*). Computed tomography re-construction images of access vessels diameter and anatomy (*E*).

Seventy-five years old frail, insulin-dependent diabetic, hypertensive female with chronic kidney disease Stage III was symptomatic with New York Heart Association (NYHA) Class III dyspnoea. She had tricuspid aortic valve, severe AS with maximum, and mean gradient of 110 and 65 mmHg and an effective orifice area of 0.6 cm^2^. Coronary evaluation revealed calcified significant distal LM and ostial left anterior descending (LAD) and borderline right coronary artery (RCA) lesions. The left ventricular systolic ejection fraction (LVEF) was 60%. With a Society of Thoracic Surgery (STS) risk score of 16 and EUROSCORE risk of in-hospital mortality of 8.25%, she was considered high risk for surgical management. A detailed computed tomography angiogram (*Figure 1A–D*) was performed for the evaluation of a possible TAVR. It showed valve morphology and peripheral vasculature suitable for transfemoral TAVR (*Figure 1E*) and thus, she was offered an option of PCI to LM and LAD with RA and a transfemoral TAVR. As the patient was planned for both PCI and TAVR, she was loaded with Aspirin 300 mg and Clopidogrel 300 mg a day prior to the procedure.

**Figure 2:**
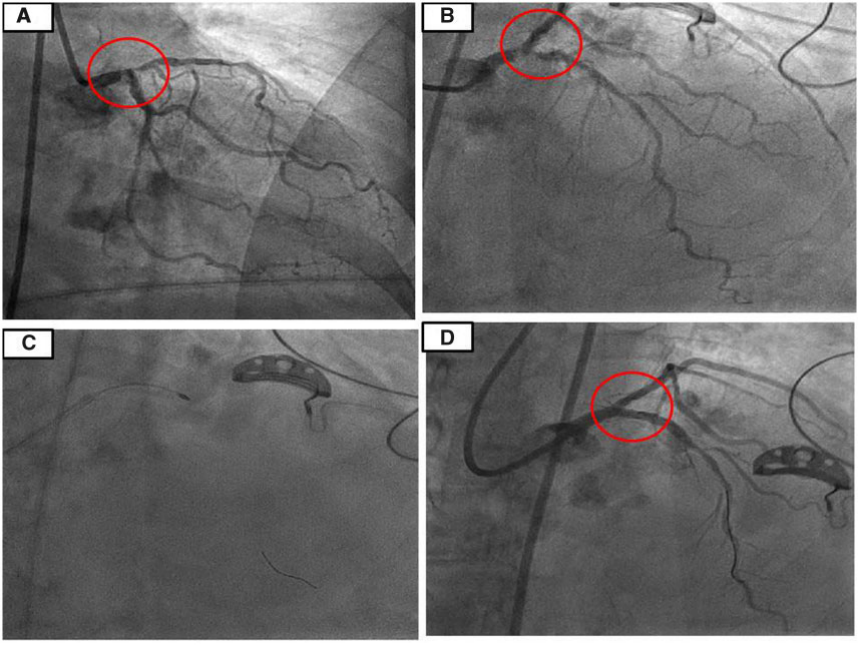
Highly calcified left main distal and ostial left anterior descending artery lesion (Medina1,1,0) [caudal and cranial views] (*A, B*). Percutaneous intervention to left main and left anterior descending artery lesion using 1.5 mm burr @1 80 000 r.p.m. (*C*). Final result after rotablation and percutaneous intervention to left main and left anterior descending artery lesion (*D*).

**Figure 3:**
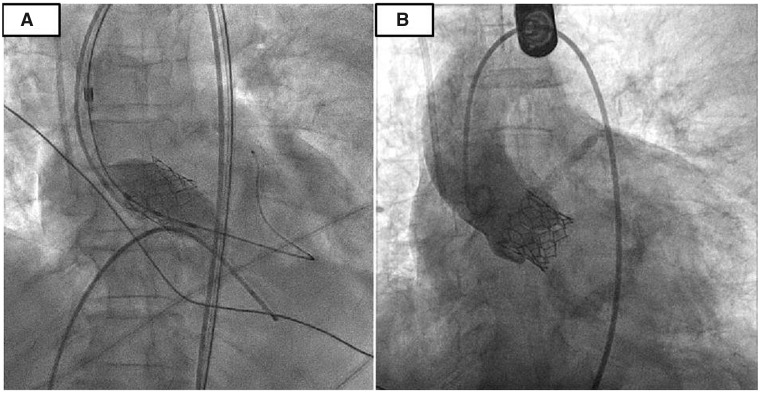
Balloon expandable Myvalv^TM^ (*A*). Myval^TM^*in situ*; no paravalvular regurgitation (*B*).

We obtained a 7 French (Fr) access in the left femoral artery for the PCI. A 7 Fr extra backup left guide catheter was used to engage LM ostium. After crossing the lesion with Sion Blue^TM^ (Asahi Intecc) guidewire, it was exchanged with RA wire over a microcatheter. A 1.5 mm burr was used for RA of LM to LAD (for Medina 1,1,0 lesion) (*Figure 2 A–C*). Following RA, the lesion was predilated with 3.0 mm non-compliant (NC) balloon and stented with a 3.5 mm × 24 mm SYNERGY^TM^ everolimus-eluting stent (Boston Scientific). Proximal optimization of stent in LM was performed with a 4.5 mm NC balloon. There was good expansion at distal LM and ostial LAD without any significant carina/plaque shift towards left circumflex (*Figure 2D*). Total fluoroscopy time was 8 min, and contrast used was only 30 mL for PCI. As the patient tolerated the procedure well with minimal contrast, we decided to go ahead with transfemoral TAVR using the right groyne as the access. Two suture based Perclose ProGlide™ devices (Abbot Vascular) were pre-deployed at 10 and 2'o clock position in the right femoral artery and a 14 Fr expandable Python^TM^ (Meril Life Sciences) sheath was inserted under fluoroscopic guidance upto the right iliac artery. Native aortic valve was dilated with a 16 mm Mammoth^TM^ balloon (Meril Life Sciences) over the Amplatz^TM^ super-stiff wire (Boston Scientific) followed by deployment of 21 mm balloon-expandable Myval^TM^ (Meril Life Sciences) (*Figure 3A, B*). Transoesophageal echocardiography showed perfect positioning and function of the valve with no paravalvular regurgitation. Right groin access was successfully closed with two pre-deployed ProGlides™. The procedure was uneventful, and the patient was discharged on Day 3 after confirmation of no new renal dysfunction or access site vascular complications. She was continued on dual antiplatelets (Aspirin and Clopidogrel) for another 6 months without any oral anticoagulants. At a follow-up of 6 months, she is doing well and is in NYHA Class I.

## Discussion

Sixty percent of patients undergoing surgical AVR (SAVR) and 40–75% of those being taken for TAVR have an associated significant CAD.^1^^,^^4^ Coronary artery bypass graft (CABG) at the time of SAVR increases the risk of short-term mortality, myocardial infarction, and stroke as compared to SAVR alone. However, this is not the case when PCI and TAVR are combined.^5^^,^^6^ Percutaneous coronary intervention with staged TAVR is increasingly being done in high surgical risk patients and with evidence from PARTNER 2 and SURTAVI,^7^^,^^8^ this strategy is comparable to surgery in intermediate-risk patients as well. However, preprocedural evaluation is of paramount importance while considering PCI along with TAVR. This includes assessment of coronary artery origin site, the height of coronaries in relation to aortic annulus, size of coronary arteries, and extent of disease involving ostium of LM or RCA.^9^^,^^10^ All these parameters need to be suitable for performing both TAVR and PCI successfully without complications. Our patient had a normal origin of coronary arteries, adequate height of LM (10 mm) and RCA (12.8 mm), and no significant disease of ostial LM artery. However, a dense calcific lesion in the distal LM and ostial LAD artery made us use the RA for providing optimal results of PCI prior to TAVR. Assessment of femoral artery anatomy is also essential as to decide which side should be used for PCI and TAVR.^10^ As our patient’s both femoral arteries were adequate size (>6 mm) for TAVR, we preferred left side for PCI and right side for TAVR based on the comfort of primary operator.

The timing of PCI in relation to TAVR is a matter of debate and the controversy is still not resolved. A staged procedure reduces the procedural time, contrast used, and risk of haemodynamic instability^5^^,^^11–13^ while a single-stage procedure appears more practical, reduces finances, and risk of another invasive procedure.^4^^,^^14^ The latest study^15^ and a recent meta-analysis^16^ have shown no difference in outcomes based on timing (before, concomitant, or after) of PCI in relation to TAVR. In our case, we planned PCI and TAVR simultaneously; as the patient had a focal coronary lesion with normal LVEF and we did not anticipate a complicated procedure of PCI. However, if during the PCI the patient would have had a haemodynamic compromise or a prolonged procedure requiring excess contrast agent, we would have deferred the TAVR to a later date.

Very few cases have been reported where a complex PCI to LM with RA and TAVR has been performed in the same setting^2^^,^^3^^,^^17^ and in all these cases, BAV was done prior to PCI to improve the cardiac output and coronary perfusion. However, aortic valve stent was placed after PCI, as engaging the coronaries may be difficult especially after the deployment of valve.^2^^,^^3^^,^^11^ We did not use BAV as a bridge to PCI as we were quite confident of successful outcome of PCI and also feared the possible complication of severe aortic regurgitation (AR) after BAV; which may force us to perform implantation of valve before PCI and then making the PCI to LM with RA much more difficult. Moreover, any patient with critical CAD may not be able to tolerate the haemodynamic effects of acute severe AR;^18^ especially if valve deployment becomes delayed during proper positioning.

To conclude in carefully selected patients: complex PCI and TAVR may be combined in a single procedure and whether PCI to be performed first or BAV, should depend on the type of coronary lesion, patient’s LVEF, and local expertise and experience.

## Lead author biography

**Figure:**
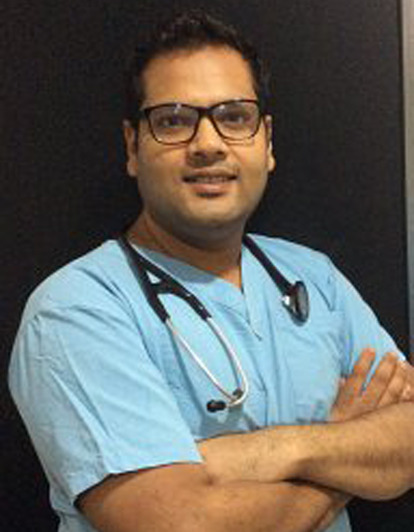


Dr Parag Barwad MD, DM, Associate Professor of Cardiology, PGIMER (Corresponding author) had special interest in structural heart disease, peripheral artery disease, and arrhythmias. 

## Supplementary material

Supplementary material is available at *European Heart Journal - Case Reports* online.

## Supplementary Material

ytaa196_Supplementary_Data
