# Supplementary material for: ROTAVI: simultaneous left main rotablation and transcutaneous aortic valve implantation in calcified coronaries and severe aortic stenosis – a case report
Source: Eur Heart J Case Rep. 2020 Aug 23;4(5):1–5. doi: 10.1093/ehjcr/ytaa196 (PMC7649449; doi:10.1093/ehjcr/ytaa196)
Supplement: ytaa196_Supplementary_Data [file ytaa196_supplementary_data.zip › ytaa196_Supplementary_Data/EHJ-CR-Slide-Set ROTAVI.pptx]

## Slide 1
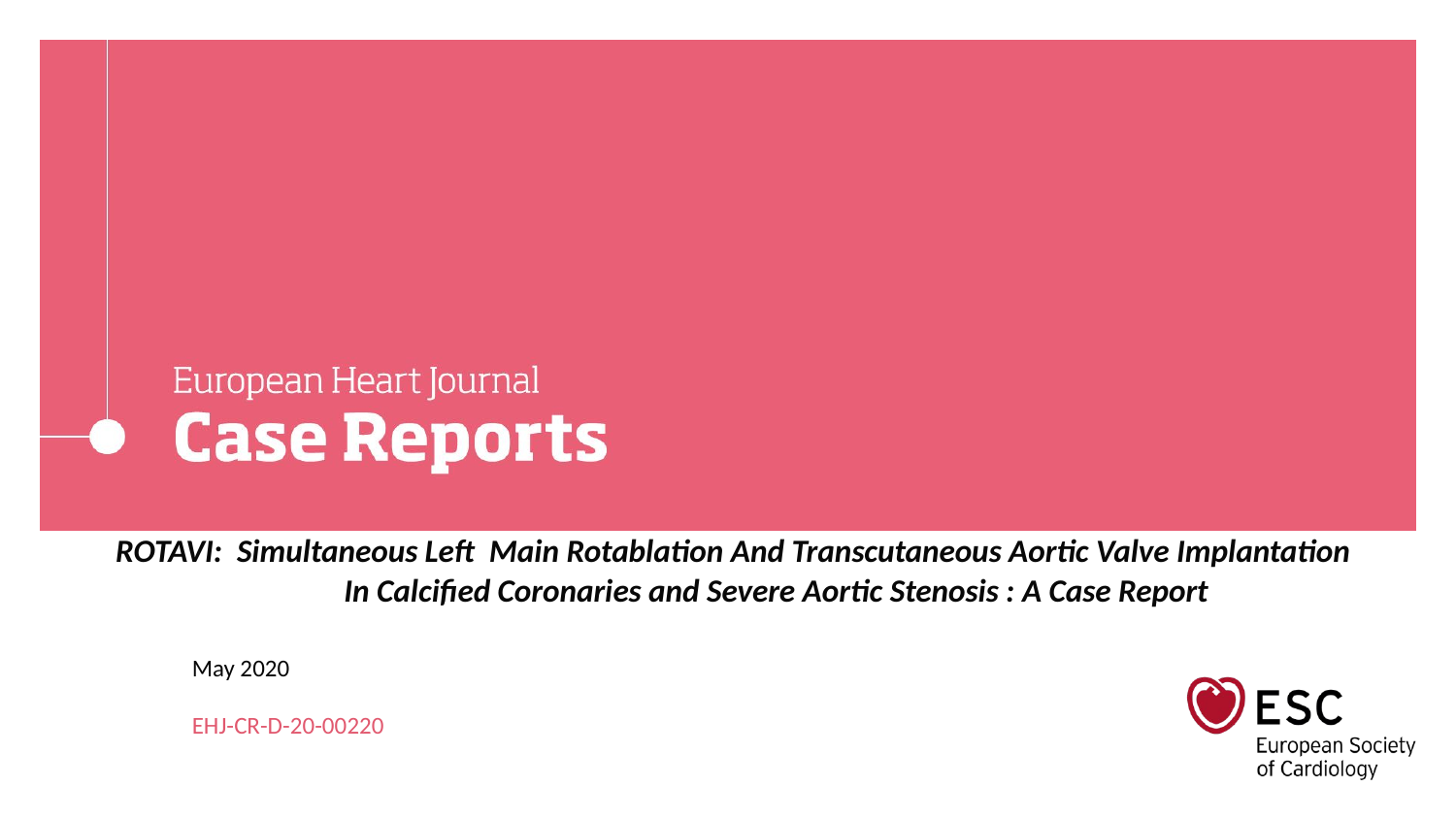

# ROTAVI: Simultaneous Left Main Rotablation And Transcutaneous Aortic Valve Implantation In Calcified Coronaries and Severe Aortic Stenosis : A Case Report
May 2020
EHJ-CR-D-20-00220

## Slide 2
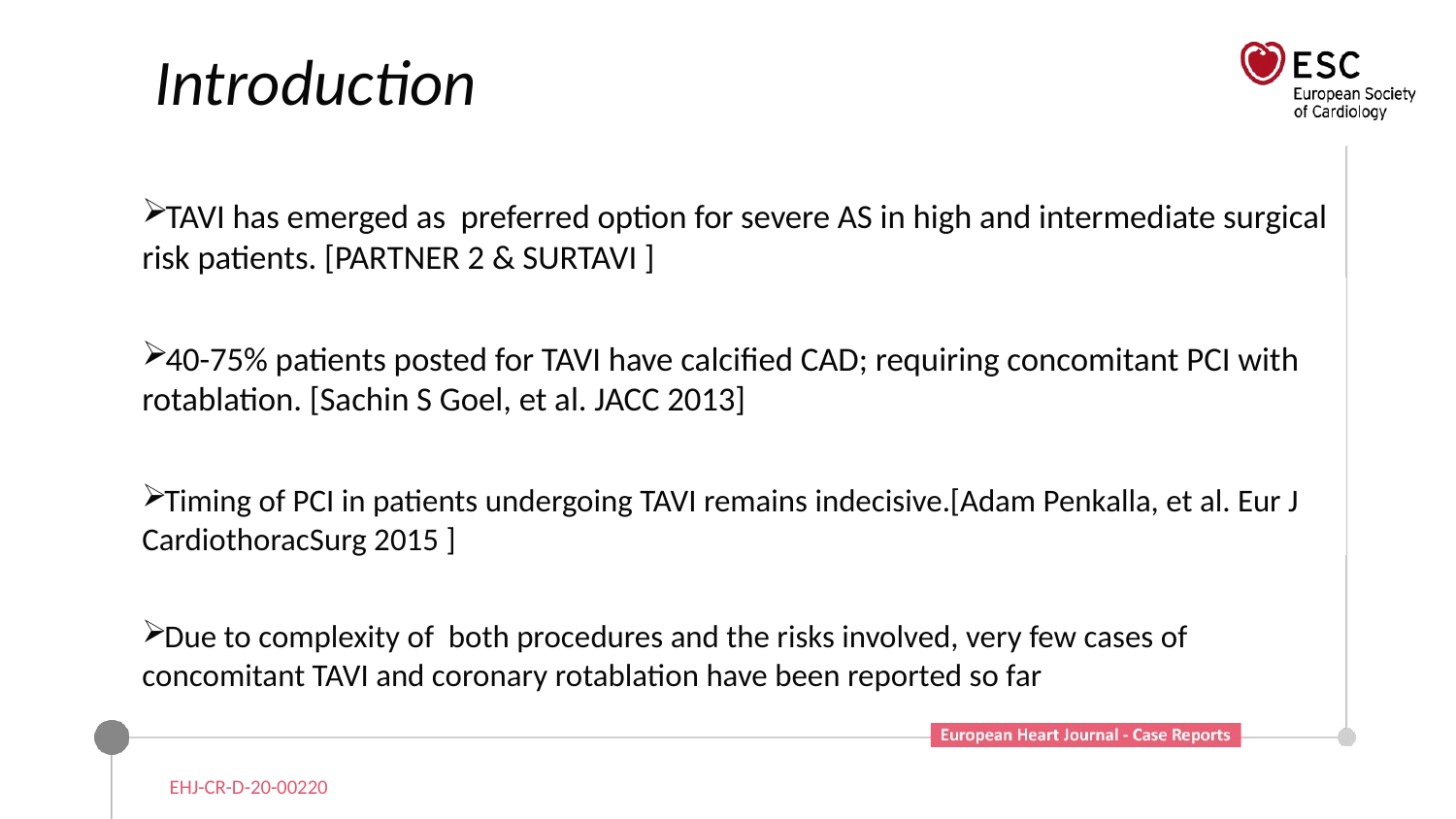

# Introduction
TAVI has emerged as preferred option for severe AS in high and intermediate surgical risk patients. [PARTNER 2 & SURTAVI ]
40-75% patients posted for TAVI have calcified CAD; requiring concomitant PCI with rotablation. [Sachin S Goel, et al. JACC 2013]
Timing of PCI in patients undergoing TAVI remains indecisive.[Adam Penkalla, et al. Eur J CardiothoracSurg 2015 ]
Due to complexity of both procedures and the risks involved, very few cases of concomitant TAVI and coronary rotablation have been reported so far
EHJ-CR-D-20-00220

## Slide 3
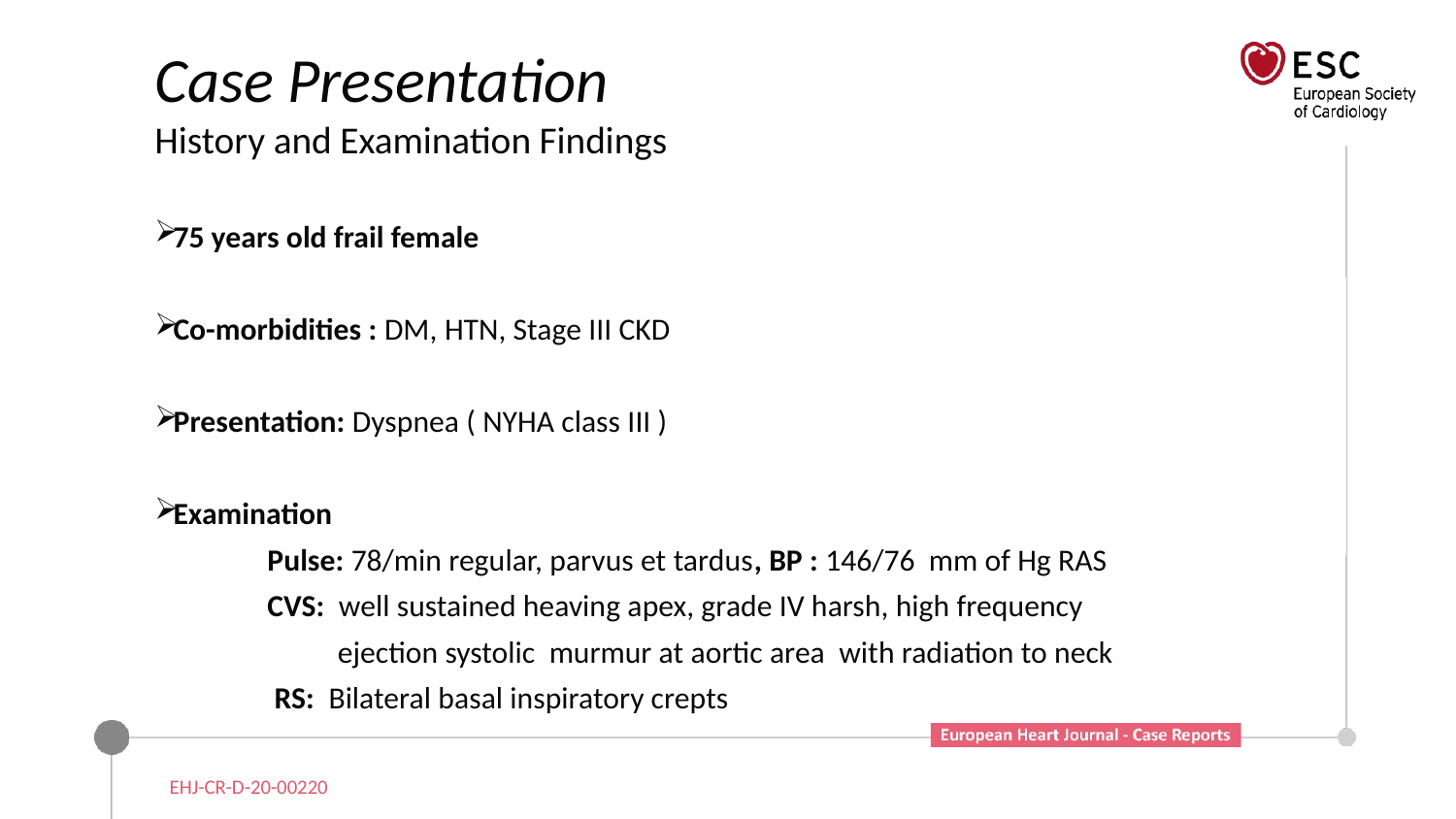

# Case Presentation History and Examination Findings
75 years old frail female
Co-morbidities : DM, HTN, Stage III CKD
Presentation: Dyspnea ( NYHA class III )
Examination
 Pulse: 78/min regular, parvus et tardus, BP : 146/76 mm of Hg RAS
 CVS: well sustained heaving apex, grade IV harsh, high frequency
 ejection systolic murmur at aortic area with radiation to neck
 RS: Bilateral basal inspiratory crepts
EHJ-CR-D-20-00220

## Slide 4
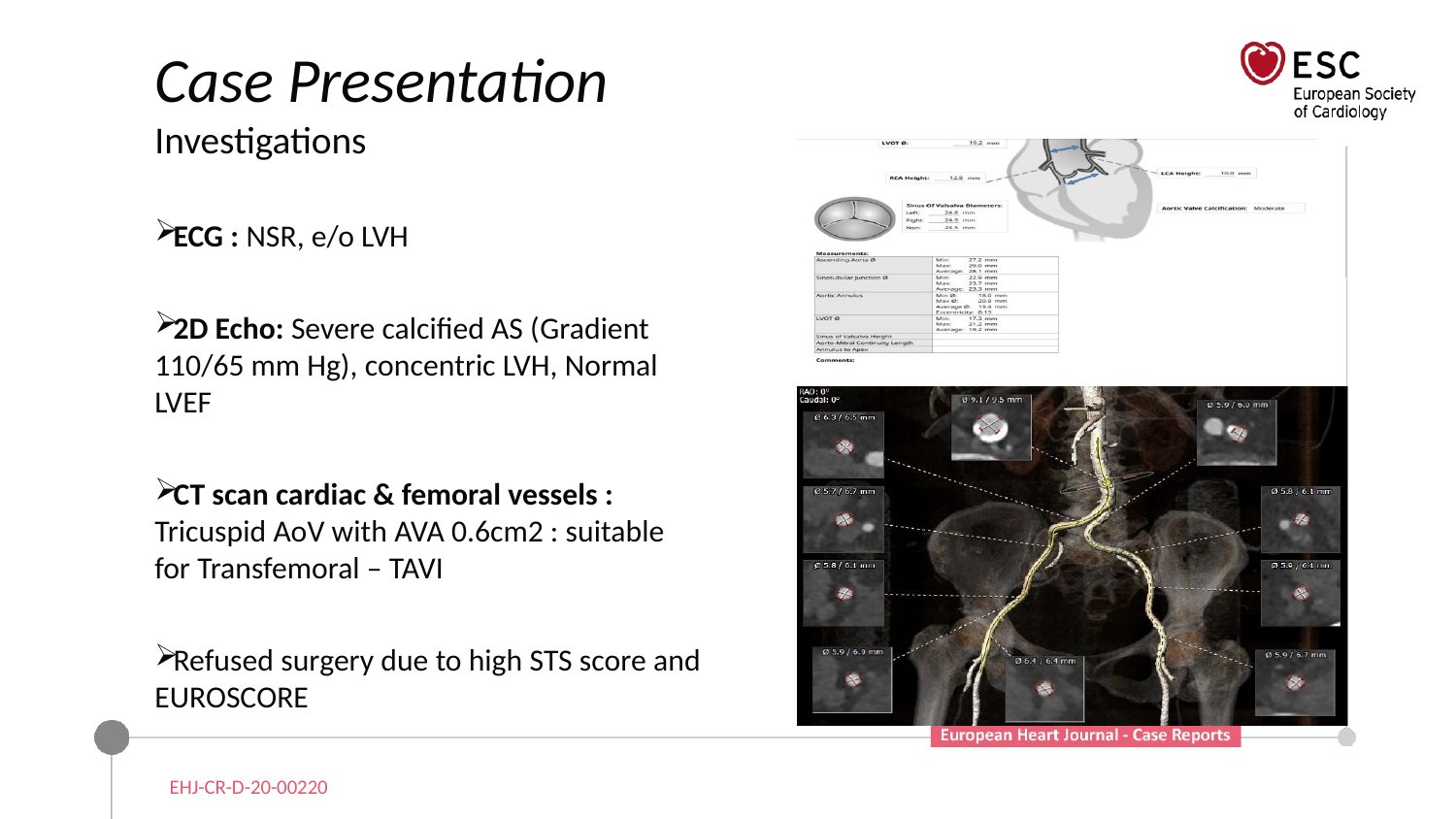

# Case Presentation Investigations
ECG : NSR, e/o LVH
2D Echo: Severe calcified AS (Gradient 110/65 mm Hg), concentric LVH, Normal LVEF
CT scan cardiac & femoral vessels : Tricuspid AoV with AVA 0.6cm2 : suitable for Transfemoral – TAVI
Refused surgery due to high STS score and EUROSCORE
EHJ-CR-D-20-00220

## Slide 5
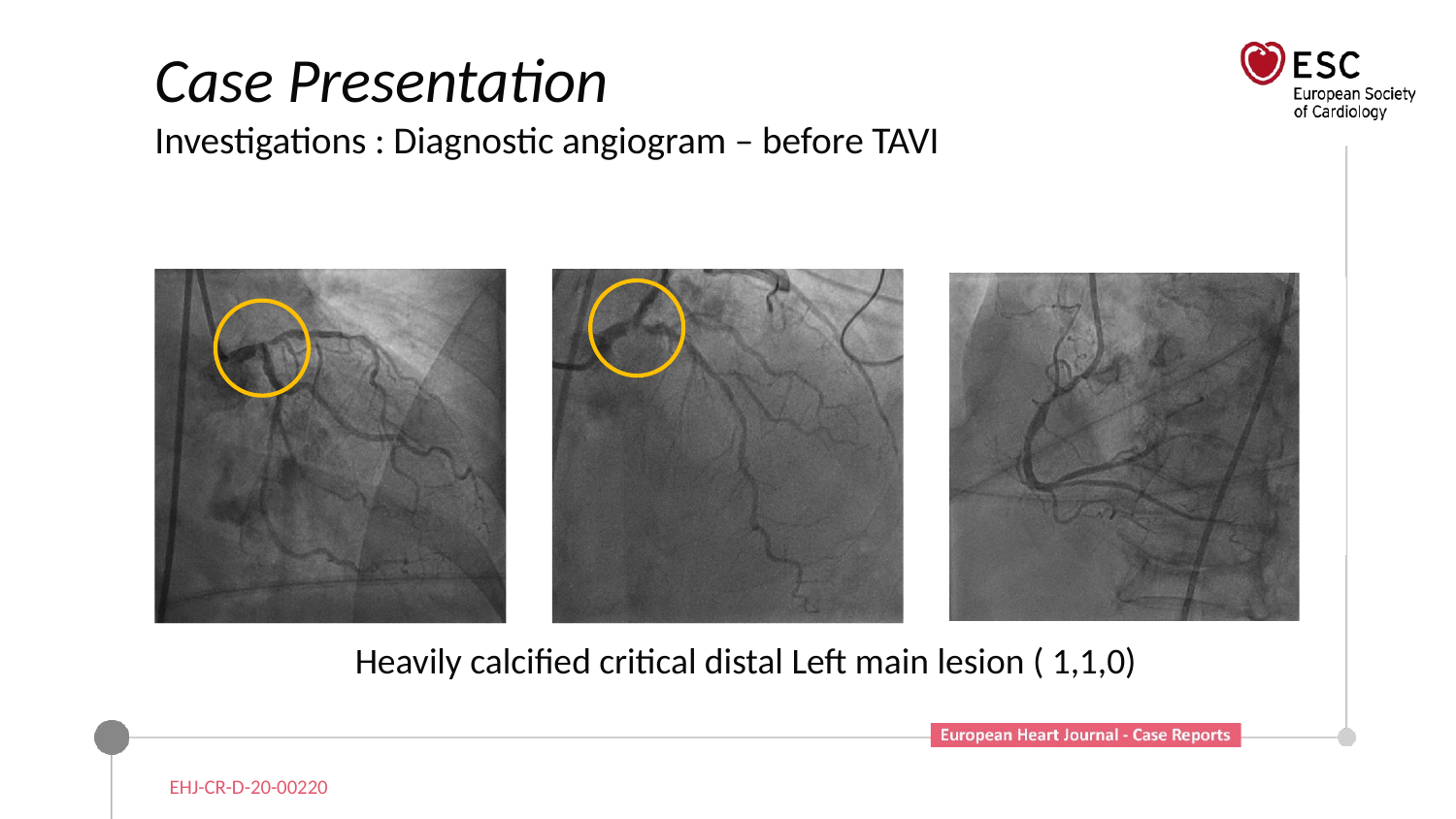

# Case Presentation Investigations : Diagnostic angiogram – before TAVI
Heavily calcified critical distal Left main lesion ( 1,1,0)
EHJ-CR-D-20-00220

## Slide 6
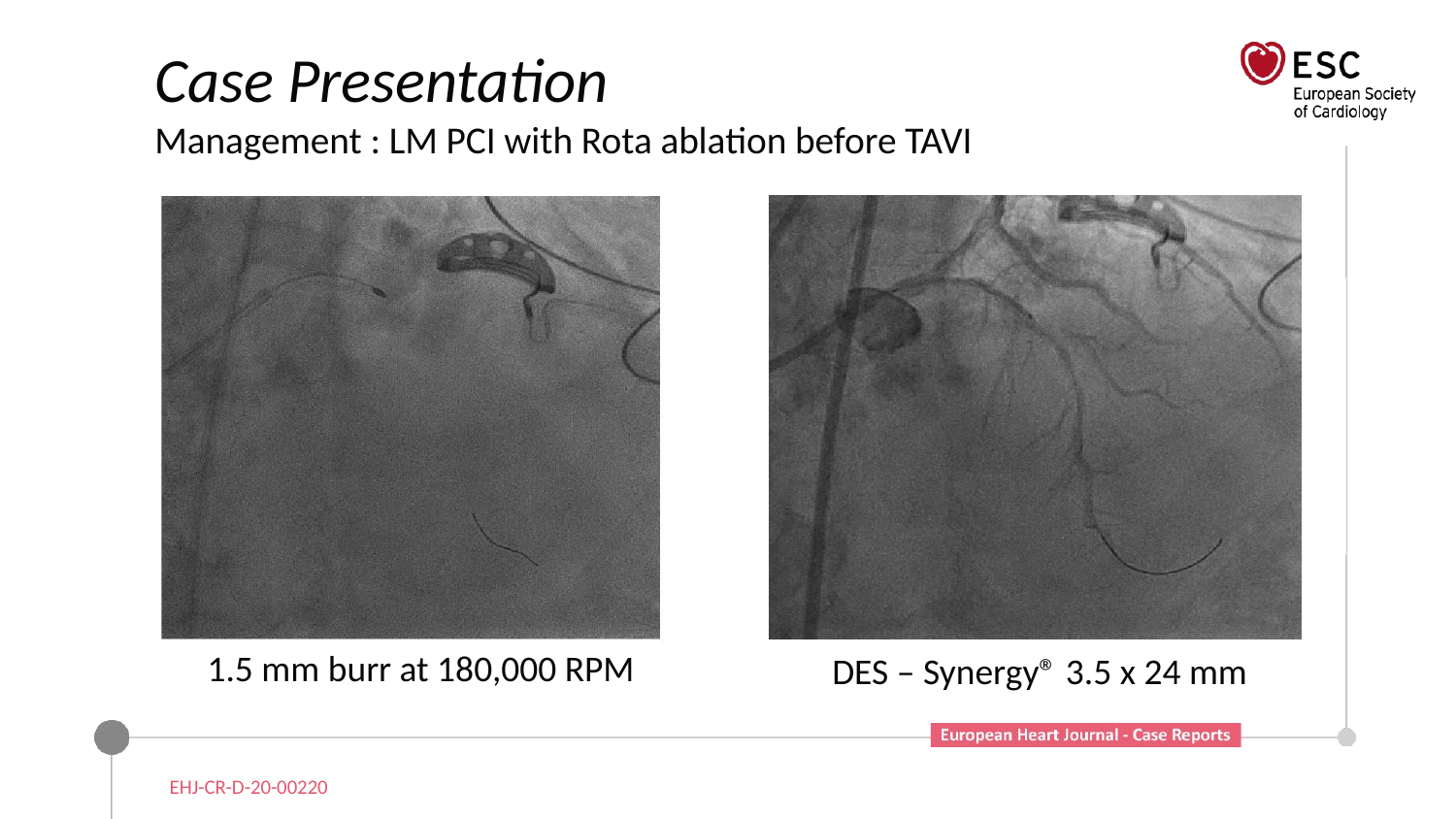

# Case Presentation Management : LM PCI with Rota ablation before TAVI
1.5 mm burr at 180,000 RPM
DES – Synergy® 3.5 x 24 mm
EHJ-CR-D-20-00220

## Slide 7
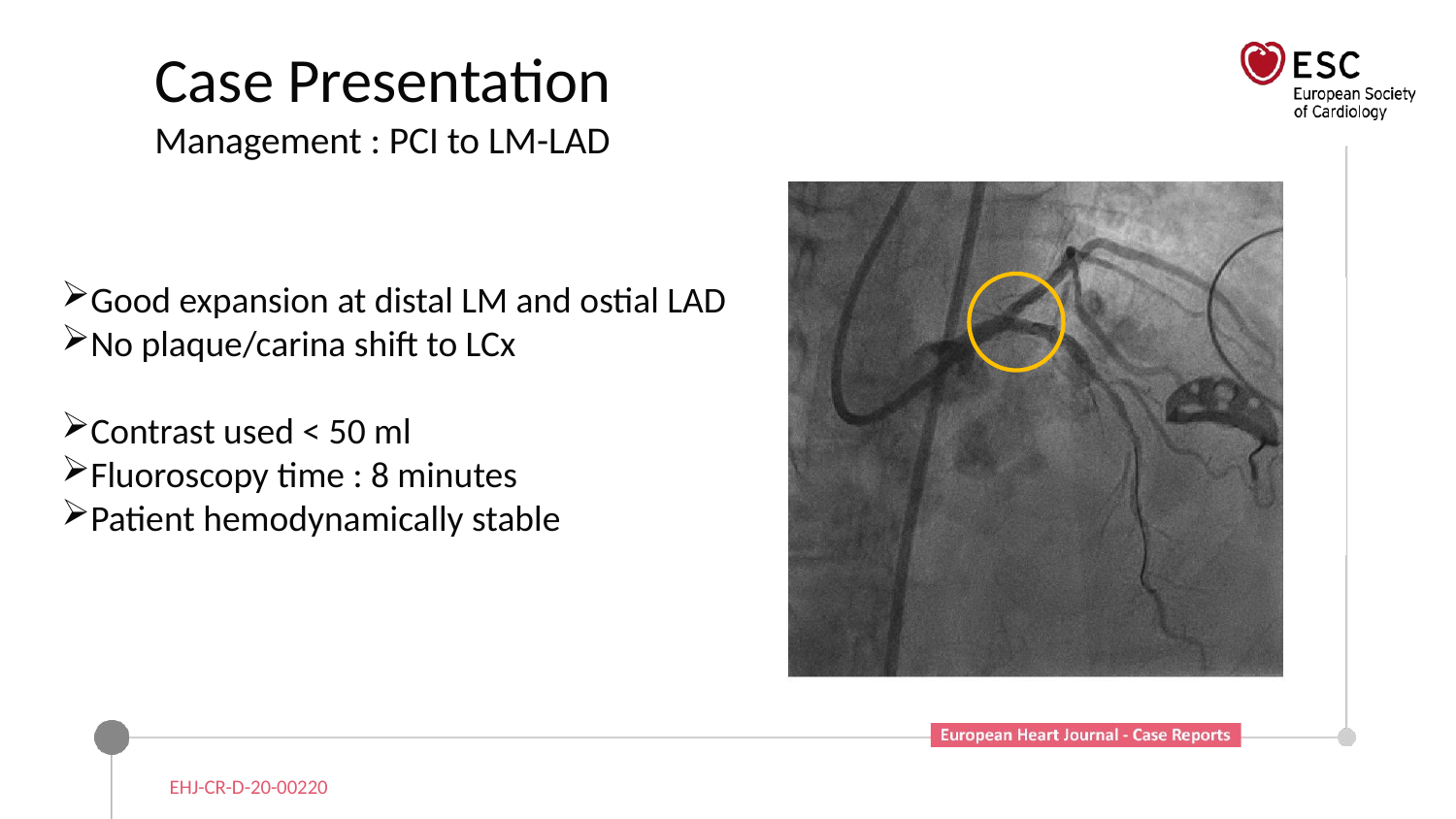

# Case Presentation Management : PCI to LM-LAD
Good expansion at distal LM and ostial LAD
No plaque/carina shift to LCx
Contrast used < 50 ml
Fluoroscopy time : 8 minutes
Patient hemodynamically stable
EHJ-CR-D-20-00220

## Slide 8
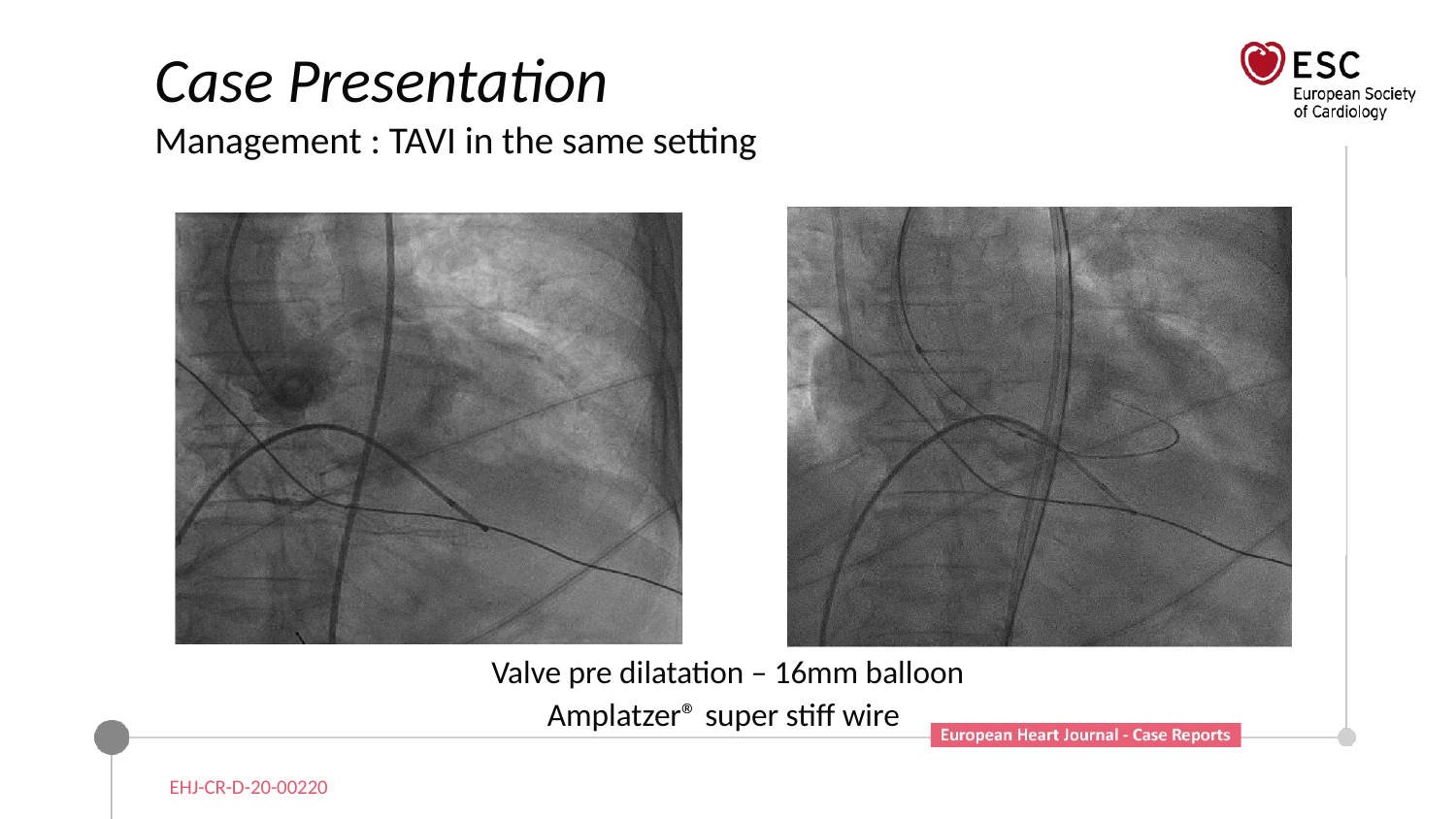

# Case Presentation Management : TAVI in the same setting
Valve pre dilatation – 16mm balloon
Amplatzer® super stiff wire
EHJ-CR-D-20-00220

## Slide 9
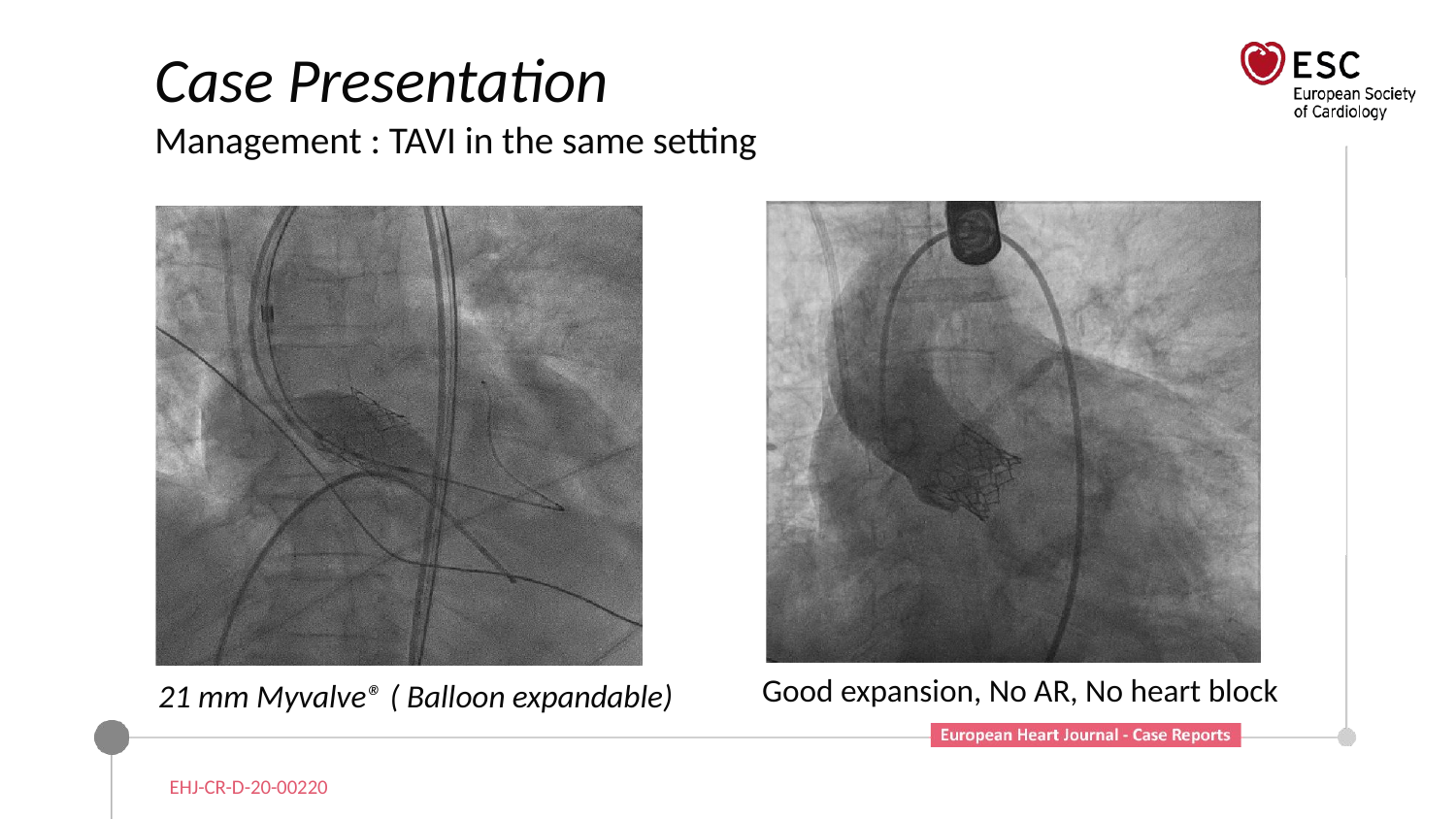

# Case Presentation Management : TAVI in the same setting
Good expansion, No AR, No heart block
21 mm Myvalve® ( Balloon expandable)
EHJ-CR-D-20-00220

## Slide 10
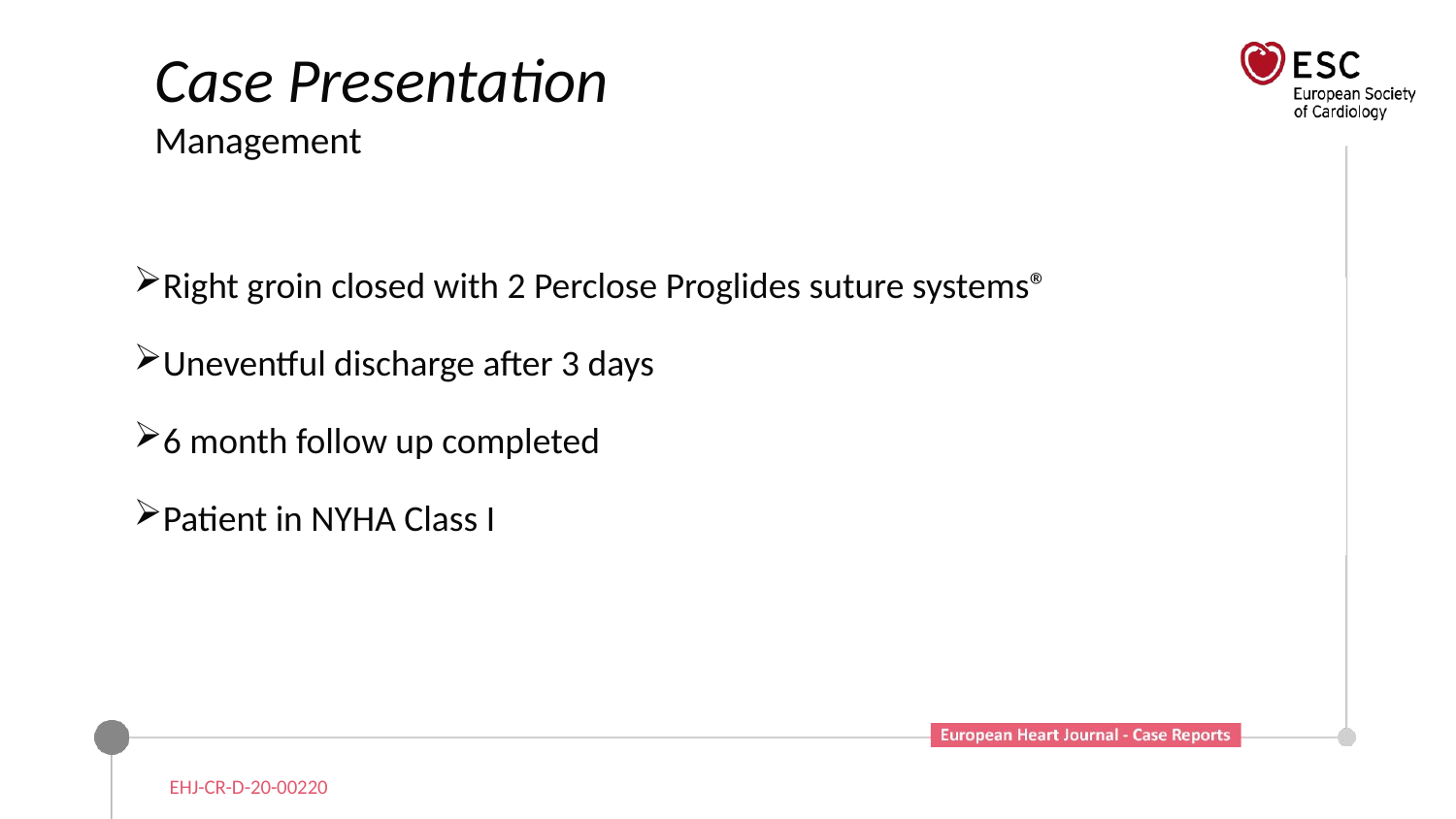

# Case Presentation Management
Right groin closed with 2 Perclose Proglides suture systems®
Uneventful discharge after 3 days
6 month follow up completed
Patient in NYHA Class I
EHJ-CR-D-20-00220

## Slide 11
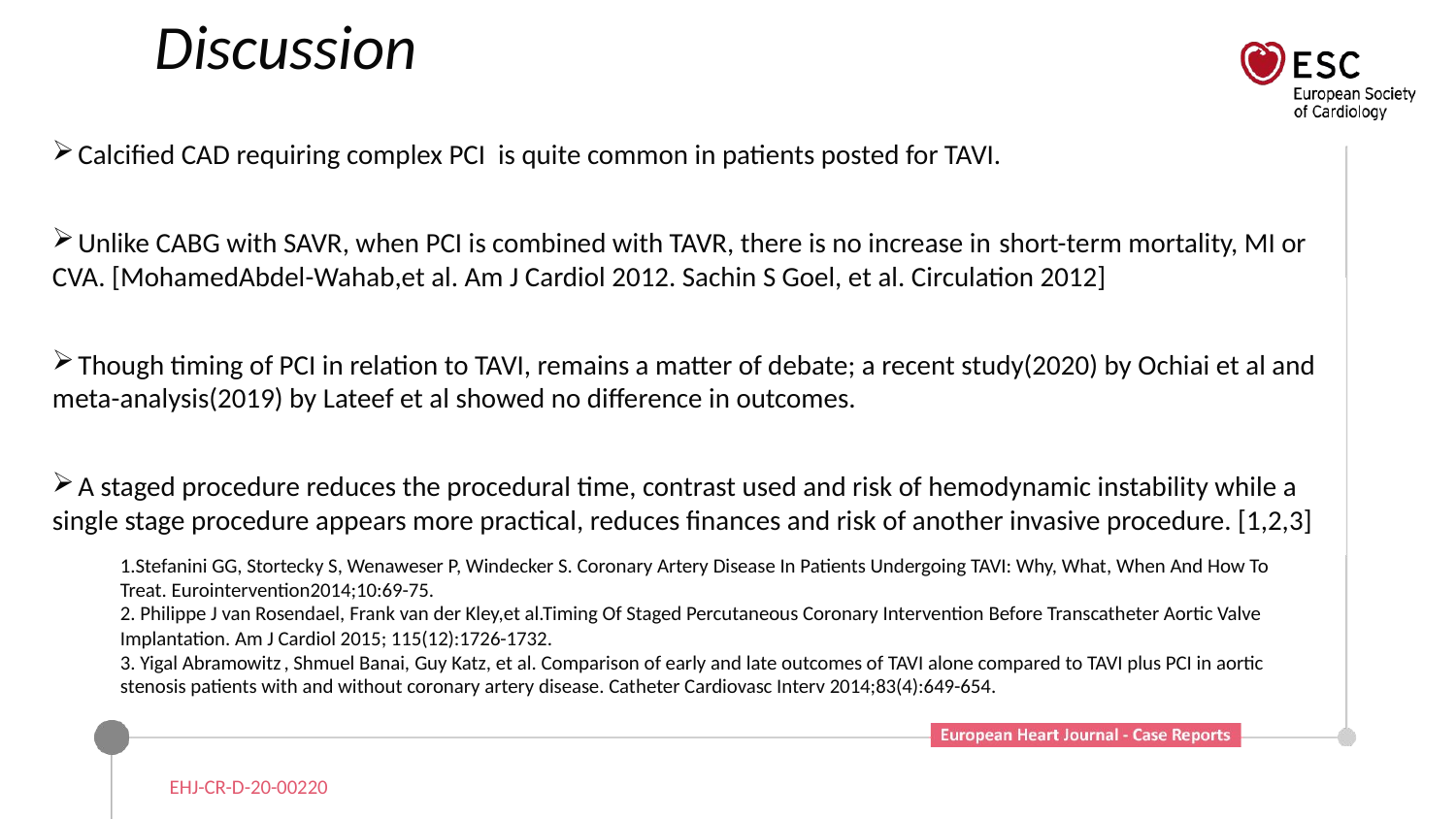

# Discussion
 Calcified CAD requiring complex PCI is quite common in patients posted for TAVI.
 Unlike CABG with SAVR, when PCI is combined with TAVR, there is no increase in short-term mortality, MI or CVA. [MohamedAbdel-Wahab,et al. Am J Cardiol 2012. Sachin S Goel, et al. Circulation 2012]
 Though timing of PCI in relation to TAVI, remains a matter of debate; a recent study(2020) by Ochiai et al and meta-analysis(2019) by Lateef et al showed no difference in outcomes.
 A staged procedure reduces the procedural time, contrast used and risk of hemodynamic instability while a single stage procedure appears more practical, reduces finances and risk of another invasive procedure. [1,2,3]
1.Stefanini GG, Stortecky S, Wenaweser P, Windecker S. Coronary Artery Disease In Patients Undergoing TAVI: Why, What, When And How To Treat. Eurointervention2014;10:69-75.
2. Philippe J van Rosendael, Frank van der Kley,et al.Timing Of Staged Percutaneous Coronary Intervention Before Transcatheter Aortic Valve Implantation. Am J Cardiol 2015; 115(12):1726-1732.
3. Yigal Abramowitz , Shmuel Banai, Guy Katz, et al. Comparison of early and late outcomes of TAVI alone compared to TAVI plus PCI in aortic stenosis patients with and without coronary artery disease. Catheter Cardiovasc Interv 2014;83(4):649-654.
EHJ-CR-D-20-00220

## Slide 12
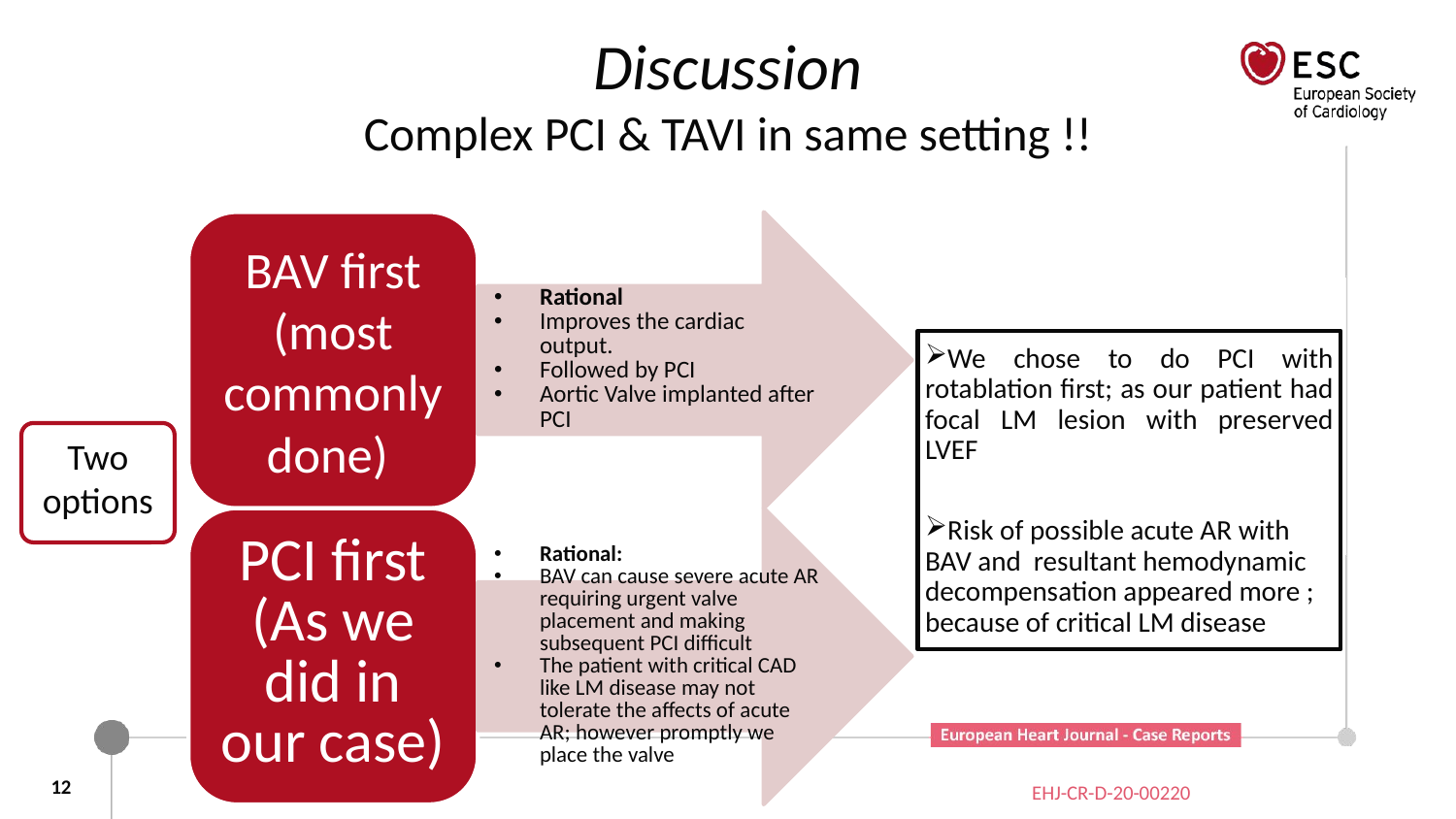

# Discussion Complex PCI & TAVI in same setting !!
We chose to do PCI with rotablation first; as our patient had focal LM lesion with preserved LVEF
Risk of possible acute AR with BAV and resultant hemodynamic decompensation appeared more ; because of critical LM disease
Two options
12
EHJ-CR-D-20-00220

## Slide 13
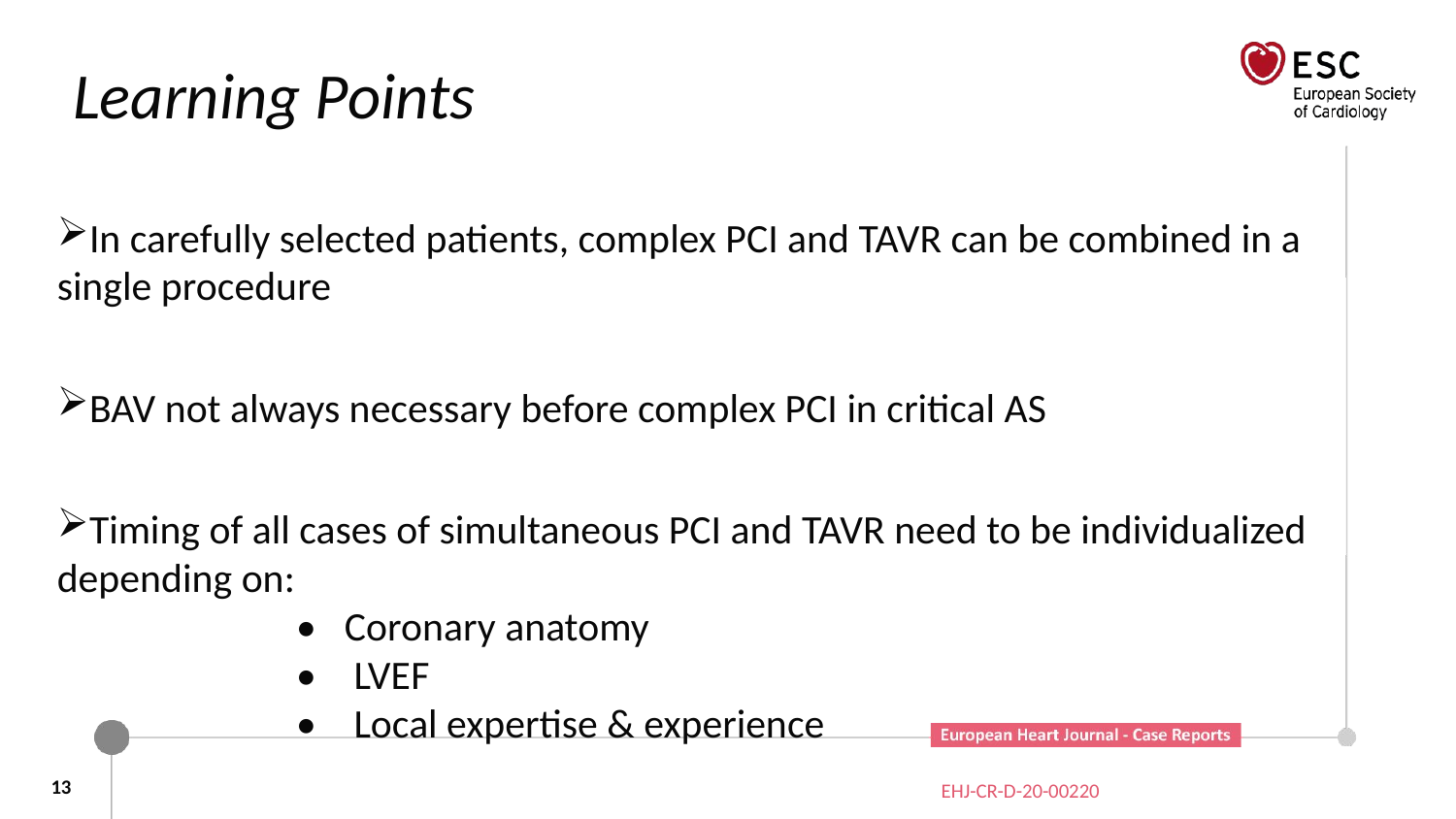

# Learning Points
In carefully selected patients, complex PCI and TAVR can be combined in a single procedure
BAV not always necessary before complex PCI in critical AS
Timing of all cases of simultaneous PCI and TAVR need to be individualized depending on:
 • Coronary anatomy
 • LVEF
 • Local expertise & experience
13
EHJ-CR-D-20-00220

## Slide 14
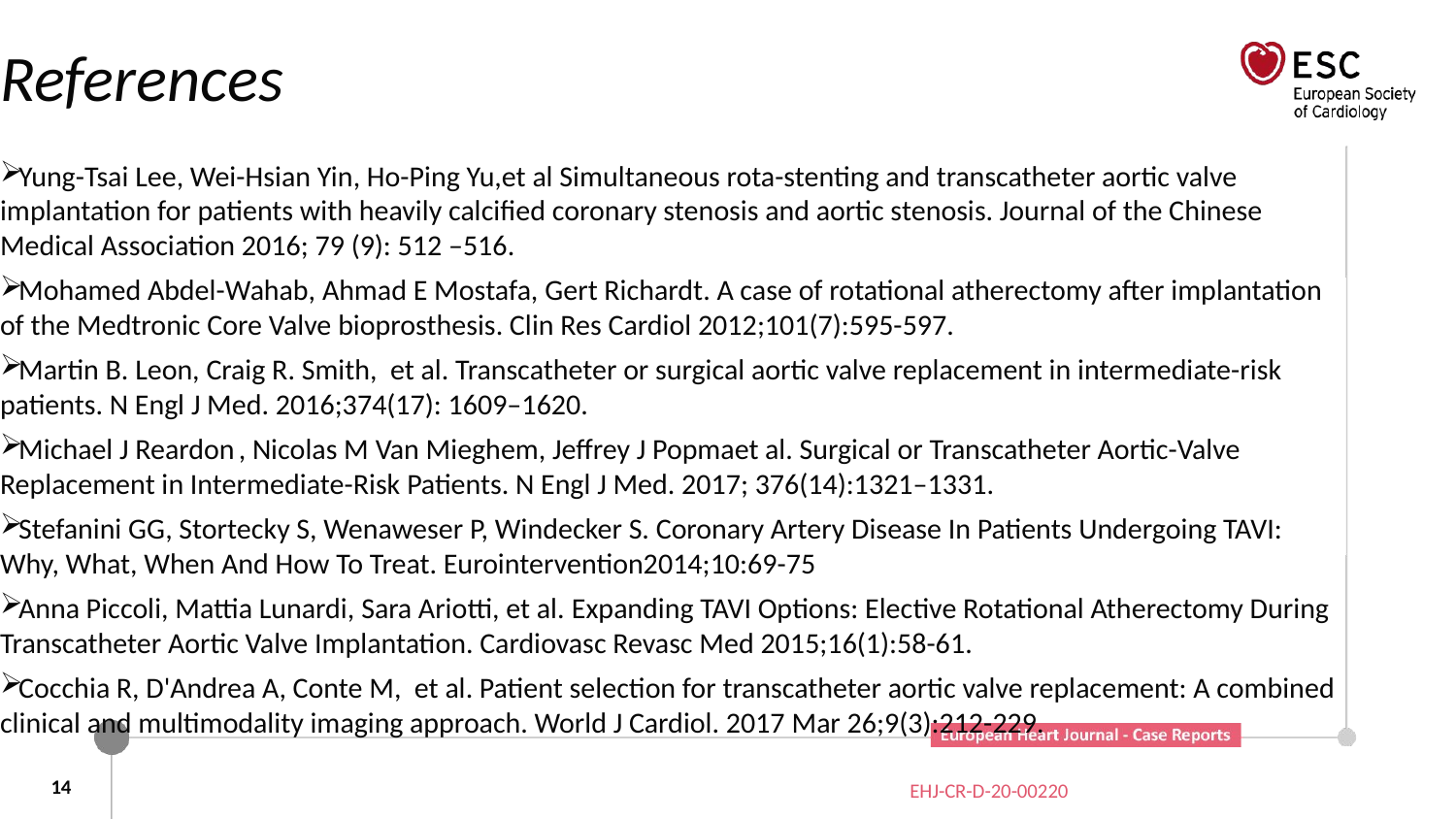

# References
Yung-Tsai Lee, Wei-Hsian Yin, Ho-Ping Yu,et al Simultaneous rota-stenting and transcatheter aortic valve implantation for patients with heavily calcified coronary stenosis and aortic stenosis. Journal of the Chinese Medical Association 2016; 79 (9): 512 –516.
Mohamed Abdel-Wahab, Ahmad E Mostafa, Gert Richardt. A case of rotational atherectomy after implantation of the Medtronic Core Valve bioprosthesis. Clin Res Cardiol 2012;101(7):595-597.
Martin B. Leon, Craig R. Smith, et al. Transcatheter or surgical aortic valve replacement in intermediate-risk patients. N Engl J Med. 2016;374(17): 1609–1620.
Michael J Reardon , Nicolas M Van Mieghem, Jeffrey J Popmaet al. Surgical or Transcatheter Aortic-Valve Replacement in Intermediate-Risk Patients. N Engl J Med. 2017; 376(14):1321–1331.
Stefanini GG, Stortecky S, Wenaweser P, Windecker S. Coronary Artery Disease In Patients Undergoing TAVI: Why, What, When And How To Treat. Eurointervention2014;10:69-75
Anna Piccoli, Mattia Lunardi, Sara Ariotti, et al. Expanding TAVI Options: Elective Rotational Atherectomy During Transcatheter Aortic Valve Implantation. Cardiovasc Revasc Med 2015;16(1):58-61.
Cocchia R, D'Andrea A, Conte M, et al. Patient selection for transcatheter aortic valve replacement: A combined clinical and multimodality imaging approach. World J Cardiol. 2017 Mar 26;9(3):212-229.
14
EHJ-CR-D-20-00220
